# Monitoring treatment of delayed cerebral ischaemia in unconscious patients after aneurysmal subarachnoid haemorrhage: a prospective multimodal neuromonitoring study

**DOI:** 10.1136/svn-2025-004642

**Published:** 2025-11-04

**Authors:** Michael Veldeman, Stefan Yu Bögli, Ihsane Olakorede, Nick Kastenholz, Miriam Weiss, Catharina Conzen-Dilger, Katharina Sophie Seyfried, Erta Beqiri, Charlotte S Weyland, Hans Clusmann, Gerrit A Schubert, Anke Hoellig, Peter Smielewski

**Affiliations:** 1 Department of Neurosurgery, RWTH Aachen University, Aachen, Germany; 2 Department of Clinical Neurosciences, University of Cambridge, Cambridge, UK; 3 Department of Neurology, University of Zurich, Zürich, Switzerland; 4 University of Cambridge, Cambridge, UK; 5 Department of Neurosurgery, University of Cologne, Cologne, Germany; 6 Department of Neurosurgery, Cantonal Hospital Aarau, Aarau, Switzerland; 7 University of Bern, Bern, Switzerland; 8 RWTH Aachen University, Aachen, Germany; 9 Department of Diagnostic and Interventional Neuroradiology, RWTH Aachen University, Aachen, Germany

**Keywords:** Aneurysm, Cerebral Infarction, Hemorrhage, Intracranial Aneurysm, Subarachnoid

## Abstract

**Background:**

Aneurysmal subarachnoid haemorrhage (SAH) is a life-threatening condition with high morbidity. Delayed cerebral ischaemia (DCI) significantly contributes to secondary injury and poor outcomes. While perfusion CT (CTP) aids DCI detection, multimodal neuromonitoring—including brain tissue oxygenation (PtiO_2_) and cerebral microdialysis—offers superior temporal resolution. Its value in guiding treatment remains underexplored.

**Methods:**

This prospective cohort study included SAH patients monitored with multimodal neuromonitoring at RWTH Aachen University Hospital (2014–2020). DCI was diagnosed with neuromonitoring abnormalities and confirmed by CTP. First-line treatment involved induced hypertension, with endovascular rescue treatment for refractory cases. Physiological data were time-aligned to treatment onset and aggregated into hourly summaries. Binomial logistic regression identified predictors of favourable 1-year outcome (modified Rankin Scale 0–3).

**Results:**

Of 56 patients with confirmed DCI, correctly placed probes and available outcome data, 22 (39.3%) achieved favourable outcome. These patients showed greater post-treatment reductions in pressure reactivity index (PRx) and lactate-to-pyruvate ratio (LPR). In multivariable analysis, greater PRx reduction was significantly associated with favourable outcome (OR 0.023, 95% CI 0.001 to 0.394, p=0.009), translating to a 46.5% increase in odds per 0.1-unit decrease. Greater LPR reductions were also predictive (OR 0.950, 95% CI 0.904 to 0.998, p=0.042), with a 5-unit drop linked to a 29.3% increase in odds. Although PtiO2_₂_ improved post-treatment, it was not associated with outcome.

**Conclusion:**

PRx and LPR reflect meaningful physiological responses to DCI treatment and are associated with 1-year outcomes. Multimodal neuromonitoring may support not only diagnosis but also treatment monitoring and decision-making in unconscious SAH patients.

**Trial registration number:**

German Clinical Trial Registry (DRKS00030505).

**WHAT IS ALREADY KNOWN ON THIS TOPIC**

- Multimodal neuromonitoring, including brain tissue oxygenation and cerebral microdialysis, offers high temporal resolution for detecting delayed cerebral ischaemia. While these tools support diagnosis, their role in guiding treatment and predicting outcomes remains unclear.

**WHAT THIS STUDY ADDS**

- This study demonstrates that reductions in pressure reactivity index (PRx) and lactate-to-pyruvate ratio (LPR) after induced hypertension are significantly associated with favourable 1-year functional outcomes in subarachnoid haemorrhage (SAH) patients. In contrast, increases in PtiO_2_ were not predictive of outcome.

**HOW THIS STUDY MIGHT AFFECT RESEARCH, PRACTICE OR POLICY**

- These findings support the use of PRx and LPR as responsive markers of treatment success in unconscious SAH patients, potentially guiding individualised therapeutic decisions when clinical examination is not feasible. This expands the role of multimodal neuromonitoring from diagnostic monitoring to dynamic treatment assessment.

## Introduction

 Aneurysmal subarachnoid haemorrhage (SAH) is a devastating disease affecting approximately 9 in 100 000 individuals annually.^1 2^ By predominantly occurring in individuals in the prime of their lives (typically in their fourth or fifth decade), SAH imposes a socioeconomic burden comparable to that of the more prevalent ischaemic stroke.^3^ The initial aneurysm rupture causes a sharp increase in intracranial pressure (ICP), resulting in a reduction in cerebral perfusion pressure (CPP). This acute event can be fatal, with out-of-hospital mortality rates reported around 15%.^4^

Following intensive care unit (ICU) admission, patients remain at risk of secondary brain injury due to delayed cerebral ischaemia (DCI). While previously attributed primarily to angiographic vasospasm, DCI is now understood as a multifactorial process.^5 6^ If left undiagnosed, untreated or if treatment is unsuccessful, the resulting injury may become irreversible in the form of DCI-related infarction. The latter is the most critical determinant of functional outcome following aneurysmal SAH.^7^

Diagnosing DCI in unconscious patients remains challenging. Some patients remain unconscious since onset, due to the severity of early brain injury (EBI), while others lose the ability to be neurologically assessed, for various reasons (eg, need for sedation, DCI). Perfusion CT (CTP) has emerged as a valuable tool for identifying tissue at risk before infarction occurs.^8 9^ However, diagnostic algorithms relying on fixed scanning time points (eg, days 4 and 7) often fail to detect hypoperfusion early enough.^10^ In contrast, multimodal neuromonitoring (MMM) techniques, such as invasive brain tissue oxygen measurement (PtiO_2_) and cerebral microdialysis, offer superior temporal resolution.^11^ However, their usefulness is limited as they only provide information from the region where the monitoring probe is placed (low spatial resolution). A diagnostic strategy combining the strengths of both techniques has proven beneficial in detecting DCI in time.^12^ Aberrant PtiO_2_ measurements or an elevated lactate-to-pyruvate ratio (LPR) can serve as triggers for CTP, potentially confirming DCI and delineating the approximate size and location of brain tissue at risk.

Despite negative results from a single randomised trial,^13^ compiled observational data continue to support induced hypertension (iHTN) as a first-line treatment once DCI is diagnosed.^14^ In cases of refractory DCI, endovascular rescue treatment (ERT) is an option that generally achieves good angiographic resolution of vasospasm with a relatively low risk, particularly in well-selected patient populations.^15^

However, once treatment is initiated in unconscious SAH patients, it remains unclear how treatment intensity—such as blood pressure targets—should be adjusted. Furthermore, guidance on the appropriate strategy for treatment weaning in the absence of a reliable neurological examination is lacking.

### Objectives

This study aims to identify which direct or indirect measures of cerebral metabolism, perfusion or autoregulatory status best reflect a treatment response that is associated with an improvement of clinical outcomes.

## Methods

### Participants

Since 2014, a prospective registry at RWTH Aachen University Hospital has included all consecutive patients with aneurysmal subarachnoid haemorrhage, confirmed by CT or catheter angiography. The study protocol was registered in the German Clinical Trial Registry (DRKS00030505). This cohort has been used in previous single- and multi-centre analyses.^16–20^

### Standardised treatment algorithm

Our institutional treatment protocol has been published in detail previously.^12 21^ In summary, aneurysms were secured within 24 hours via either clip ligation or endovascular embolisation, and all patients received prophylactic oral nimodipine. If acute hydrocephalus was present, it was managed by placement of an external ventricular drainage. Initial clinical severity was assessed using the best Glasgow Coma Scale score within 24 hours of admission^22^ and converted to a World Federation of Neurosurgical Societies (WFNS) grade,^23^ which was dichotomised into poor-grade (WFNS 4–5) and good-grade (WFNS 1–3) SAH. Radiological severity was evaluated using the modified Fisher scale.^24^ Patients who remained neurologically not assessable after a wake-up test, or lost the ability for neurological examination due to secondary deterioration, underwent invasive neuromonitoring. This determination of neurological assessability was made pragmatically at the bedside and was not directly related to the initial WFNS grading. Microdialysis catheters (71 High Cut-Off Brain Microdialysis Catheter, μdialysis, Sweden) and brain tissue oxygenation (PtiO_2_) probes (Neurovent PTO, Raumedic) were implanted on the side of the ruptured aneurysm or, in cases of midline aneurysms, on the side with the greatest subarachnoid blood load. Probes were inserted using a double-bolt in the frontal region, approximately 1 cm lateral to Kocher’s point, positioning the tip in the frontal watershed region to cover both anterior and middle cerebral arteries’ territories.

### Design of the study

This comparative study analysed changes in physiological parameters following DCI treatment in patients with favourable and unfavourable outcomes. All data were aligned to the hour of treatment initiation (post-CTP-confirmed DCI). Core measurements from MMM and derived parameters were assessed pretreatment and post-treatment. High-frequency data were aggregated into hourly means or medians to match microdialysis sampling. These values, along with SD or IQR (Q1–Q3), were plotted over a 10-day window starting 72 hours before treatment. Locally fitted regression lines highlighted temporal trends, identifying a key analysis window from 24 hours before to 72 hours after treatment. Due to variable-specific trends, tailored time frames were used when needed. Paired analyses compared pooled patient-level means or medians between pretreatment and post-treatment periods. Changes were calculated as post-treatment *minus* pretreatment values and compared by 1-year outcome group.

### DCI diagnosis and treatment

DCI diagnosis was supported by neuromonitoring, including transcranial Doppler vasospasm (mean flow velocity >120 cm/s in the middle cerebral artery), cerebral oxygenation crises (PtiO_2_<20 mm Hg) or metabolic disturbances (LPR>40), prompting CTP imaging. Radiological DCI was confirmed by territorial or watershed perfusion deficits on CTP, defined as mean transit time >6.7 s and time-to-drain >10 s.^25^

Treatment began after radiological confirmation, with first-line therapy using norepinephrine to induce euvolemic hypertension. CTP was repeated 6–12 hours later to evaluate the response. Persistent perfusion mismatch led to second-tier endovascular therapy, including angioplasty for proximal vasospasm and intra-arterial spasmolysis for diffuse vasospasm. Treatments were reviewed daily and adjusted as needed. Other strategies, such as intravenous milrinone infusion, intrathecal nicardipine or lumbar cerebrospinal fluid drainage, were not part of our institutional protocol during the study period. The use of lumbar drains has only become routine at our centre following the publication of the EARLYDRAIN study in 2023 and was therefore not applied in this cohort.^26^

### Data collection

Arterial blood pressure was continuously monitored via an invasive arterial line with a standard pressure transducer. High-frequency waveform data for mean arterial pressure (MAP) and ICP were recorded at 100 Hz, while PtiO_2_ measurements were collected at 1 Hz. Data acquisition was initially conducted using the MPR2 logO Datalogger (Raumedic, Helmbrechts, Germany) until July 2018, after which the Moberg CNS monitor (Component Neuromonitoring System, Moberg Research, Ambler, Pennsylvania, USA) was used. Artefacts from the initial probe calibration phase were excluded from analysis.

Clinical outcome was assessed 12 months post-haemorrhage using the modified Rankin Scale (mRS),^27^ obtained through either in-person evaluations or structured telephone interviews with patients or their next of kin. Outcome was dichotomised into favourable (mRS 0–3) and unfavourable outcome (mRS 4–6).

Microdialysis catheters were continuously perfused at a rate of 0.3 µL/min using a crystalloid perfusate (CNS perfusion fluid, μdialysis). Dialysate collection vials were replaced every 3 hours, with increased sampling frequency (hourly) in response to abnormal metabolic trends. The dialysate was analysed bedside using the ISCUSflex system (μdialysis) to measure concentrations of lactate (mmol/L), pyruvate (μmol/L), glucose (mmol/L), among others.

### Data preparation

Artefact removal was performed manually, and indices were computed using ICM+ multimodal monitoring software (University of Cambridge, Cambridge Enterprise, Cambridge, UK). Cerebrovascular reactivity was assessed using the pressure reactivity index (PRx).^28^ MAP and ICP values were averaged over 10-second intervals, while PRx was computed using a moving 5-minute window with 80% overlap, based on Pearson correlation analysis. The oxygen reactivity index was calculated similarly to the moving correlation coefficient between CPP and PtiO_2_, but using longer, 60-minute, data windows.

The anatomical relationship between monitoring probes and hypoperfused brain regions was assessed. The probe was considered to be within or adjacent to the affected area if the hypoperfused region involved the ipsilateral A1, A2, M1, M4 or M5 territories, as defined by the extended Alberta Stroke Programme Early CT Score.^29 30^

### Statistical analysis

Statistical analyses and visualisations were conducted in R (V.4.4.0; www.r-project.org) via RStudio (V.2024.12.0+467), using *tidyverse*, *broom*, *car*, *DHARMa*, *gratia*, *lme4*, *MASS*, *mice*, *performance*, *rcompanion*, *smooth* and *zoo* packages. Distributions of continuous variables were assessed via histograms, Q–Q plots and, when needed, the Shapiro-Wilk test. Normally distributed data are reported as mean±SD and compared using unpaired or paired t-tests. Non-normal data are given as median and IQR (Q1–Q3) and analysed via Mann-Whitney U or Wilcoxon signed-rank tests. Multivariate imputation by chained equations was used for <5% missing data to enable paired analyses. Categorical data were reported as percentages and assessed with χ² or Fisher’s exact test.

Exploratory analysis included MAP, CPP, PRx, PtiO_2_ and cerebral microdialysis data over 10 days, starting 72 hours pretreatment, plotted as mean±SD or median with IQR. Non-linear temporal trends were modelled using Generalised Additive Models (GAMs) with cubic splines and continuous-time autoregression. Restricted maximum likelihood estimation, optimised smoothing and turning points were derived via the first derivative of the GAM function. Peaks and troughs were identified and temporally related to treatment onset. Aggregated values over the analysis window were compared across outcome groups using univariate tests.

A binomial logistic regression model was built using backward selection, starting with predictors significant in univariate analysis. Variables were removed based on likelihood ratio test (LRT) results. Multicollinearity was assessed via Variance Inflation Factor (VIF; cut-off=2.5), and model performance at each step was evaluated using Akaike Information Criterion (AIC). Only predictors that significantly improved model fit were retained. A two-sided p value <0.05 was considered statistically significant.

The manuscript adheres to the Strengthening the Reporting of Observational Studies in Epidemiology guidelines.^31^

## Results

### Patient characteristics

**Figure 1:**
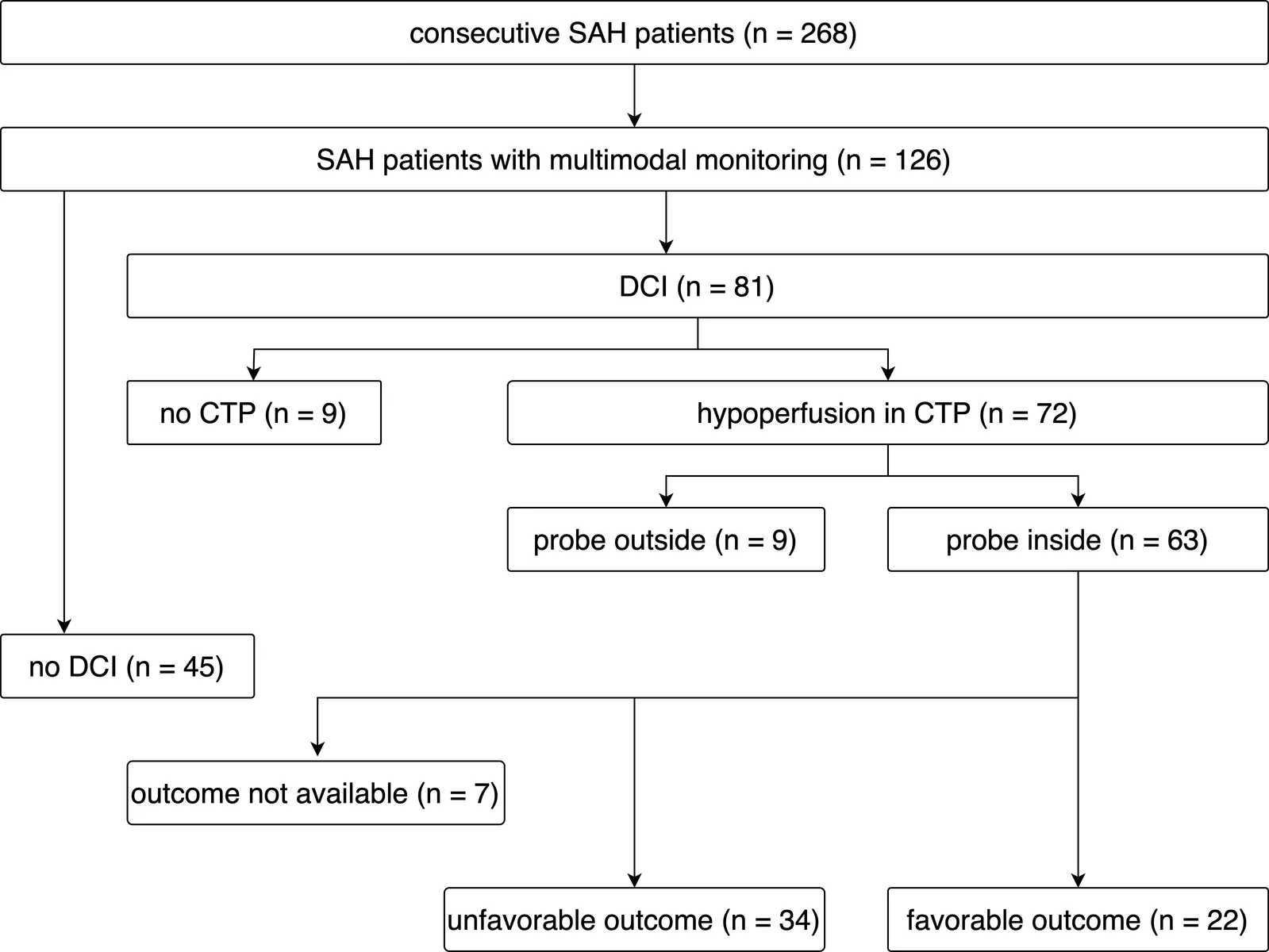
Inclusion flow chart. CTP, perfusion CT; DCI, delayed cerebral ischaemia; SAH, aneurysmal subarachnoid haemorrhage.

During the inclusion period (2014–2020), 268 patients with aneurysmal subarachnoid haemorrhage were treated at RWTH Aachen University Hospital. Due to their initial unconscious state or later loss of consciousness—whether DCI-related or not—126 patients underwent invasive multimodal neuromonitoring, including PtiO_2_ and/or cerebral microdialysis. Among these, 81 patients (64.3%) developed DCI. CTP imaging prior to treatment initiation was available for 72 patients (57.1%), and in 63 patients (50%), the invasive monitoring probe was located within or near the hypoperfused region. Out of these, all had PtiO_2_ and 51 (81%) had cerebral microdialysis. One-year outcome data were missing for seven patients (5.6%). The final analysis included 56 treated patients, of whom 34 (60.7%) had an unfavourable, while 22 (39.3%) had a favourable outcome, 1 year after aneurysm rupture. A flowchart of patient inclusion is presented in figure 1. The cohort had a mean age of 54.6±11.5 years, and 41 patients (73.2%) were female. Patient demographics, as well as haemorrhage and treatment characteristics, are detailed in table 1.

**Table 1:** Demographic and haemorrhage specific characteristics of included subarachnoid haemorrhage patients stratified by dichotomised 1-year clinical outcome

	All with outcome	Unfavourable outcome	Favourable outcome	Univariate p value
	n=56	n=34	n=22	
Demographics				
Age—years—mean±SD (range)	54.6±11.5 (19–87)	54.0±13.1 (19–87)	55.5±8.8 (35–67)	0.628
Sex—female/male—no. (%)	41 (73.2)/15 (26.8)	24 (70.6)/10 (29.4)	17 (77.3)/5 (22.7)	0.808
Monitoring				
Overall duration (days)	16.9±7.4	15.6±7.6	19.0±6.9	0.099
Lag to CTP deficit (hours)	176.0±78.1	181±72.9	167.0±86.7	0.545
Aneurysm location—no. (%)				
Ant. circulation	50 (89.3)	31 (91.2)	19 (86.4)	0.670
Diameter—mean±SD	7.0±4.1	6.4±3.2	7.5±4.6	0.290
Clipping/endovascular	20 (35.7)/36 (64.3)	17 (50.0)/17 (50.0)	3 (13.6)/19 (40.9)	**0.009**
Haemorrhage severity				
WFNS grade—no. (%)				0.349
Grade 1	7 (12.5)	4 (11.8)	3 (13.6)	
Grade 2	5 (8.9)	1 (2.9)	4 (18.2)	
Grade 3	15 (26.8)	10 (29.4)	5 (22.7)	
Grade 4	17 (30.4)	10 (29.4)	7 (31.8)	
Grade 5	12 (21.4)	9 (26.5)	3 (13.6)	
Poor-grade SAH (WFNS 3–5)	44 (78.6)	29 (85.3)	15 (68.2)	0.184
Modified Fisher scale—no. (%)				0.567
Grade 1	8 (14.3)	3 (8.8)	5 (22.7)	
Grade 2	8 (14.3)	5 (14.7)	3 (13.6)	
Grade 3	13 (23.2)	9 (26.5)	84 (18.2)	
Grade 4	27 (48.2)	17 (50.0)	10 (45.5)	
ICH	24 (42.9)	16 (47.1)	8 (36.4)	0.581
DCI treatment				
Induced hypertension—no. (%)	51 (91.1)	30 (88.2)	21 (95.5)	0.638
Duration (days)—mean±SD	11.1 (6.7)	9.1±5.7	13.9±7.2	**0.016**
ERT—no. (%)	43 (76.8)	16 (47.1)	18 (85.7)	0.091
Lag ERT (days)—mean±SD	8.2±4.2	8.3±4.5	8.1±3.9	0.824

Unfavourable outcome=modified Rankin scale 4, 5 and 6 after one year.

Favourable outcome=modified Rankin scale 0, 1, 2 and 3 after one year.

Significant p values (<0.05) are written in bold.

ant., anterior; CTP, perfusion CT; DCI, delayed cerebral ischaemia; ERT, endovascular rescue treatment; ICH, intracerebral haemorrhage; SAH, aneurysmal subarachnoid haemorrhage; WFNS, World Federation of Neurosurgical Societies grading.

When comparing both outcome groups, a lower rate of favourable outcomes was observed in clipped patients (13.6% vs 40.9%; p=0.009). The duration of iHTN treatment was, on average, longer in patients with a favourable 1-year outcome (13.9±7.2 vs 9.1±5.7 days; p=0.016). Rescue treatment was, on average, started 8.2±4.2 days after aneurysm rupture.

### Treatment response in multimodal monitoring

Based on exploratory plotting, a baseline time window of 24 hours before treatment initiation and a 72-hour window after was selected for comparison. As time trends of individual parameters considerably differed, a fixed time window around treatment would not always optimally represent actual changes over time. Subsequently, for PRx and LPR, a time window of 12 hours around the peak following treatment was compared with a time window of 72 hours starting 24 hours after the peak was reached.

An overview of pooled 24 hours before versus 72 hours after results of physiological parameters around the beginning of treatment is provided in table 2. The subgroup of patients with available outcomes is further analysed and stratified by outcome groups in table 3.

**Table 2:** Pooled monitoring data over 24 hours before and 72 hours after the initiation of DCI treatment

Treatment	24 hours before	72 hours after	Difference*	Univariate p value
MMM all (n=63)			After minus before	
MAP (mm Hg)—mean±SD	102.3±14.8	107.9±15.4	5.7±15.1	**0.007**
CPP (mm Hg)—mean±SD	93.5±13.2	98.4±14.9	10.5±15.6	**0.014**
PRx—mean±SD	0.10±0.27	0.17±0.16	0.05±0.23	**0.042**
PtiO_2_ (mm Hg)—mean±SD	18.3±13.4	25.0±11.7	6.6±14.3	**<0.001**
LPR—median (Q1–Q3)	30.6 (25.5–41.7)	29.2 (23.6–34.6)	*−1.0±16.2*	0.164
Glucose (mmol/L)—median (Q1–Q3)	1.01 (0.69–1.71)	1.36 (0.79–1.74)	*−0.05±0.99*	0.782

MMM all (n=63)	12 hours at peak	24–96 hours after	Difference	Univariate p value
			After minus before	
PRx—mean±SD	0.21±0.24	0.17±0.16	*−0.03±0.22*	0.137
LPR—median (Q1–Q3)	31.9 (25.6–37.7)	29.5 (26.1–37.9)	0.5±7.2	0.350

Negative differences are written in italic.

Significant p values (<0.05) are written in bold.

CPP, cerebral perfusion pressure; LPR, lactate-to-pyruvate index; MAP, mean arterial pressure; MMM, multimodal monitoring; PRx, pressure reactivity index; PtiO_2_, brain tissue partial oxygen pressure; Q1, first quartile; Q3, third quartile.

**Table 3:** Univariate and multivariate comparison of multimodal neuromonitoring data stratified by dichotomised 1-year clinical outcome

Treatment	All	Unfavourable outcome	Favourable outcome	Univariate p value*	OR for favourable outcome	95% CI	Multivariate p value†
	n=56	n=34	n=22				
MMM—hours before vs after							
Mean±SD							
MAP (mm Hg)	6.3±18.3	3.3±16.3	10.3±20.3	0.182			
CPP (mm Hg)	4.1±14.0	2.7±14.2	6.1±13.9	0.382			
PRx	0.03±0.25	0.10±0.26	−0.06±0.20	**0.018**	0.572	0.001 to 0.394	**0.009**
PtiO_2_ (mm Hg)	8.2±10.9	8.0±12.8	8.6±7.9	0.841			
LPR	*−6.8±16.5*	1.3±9.1	−17.6±18.2	**<0.001**	0.950	0.904 to 0.998	**0.042**
Glucose (mmol/L)	0.19±0.93	0.13±0.94	0.28±0.93	0.733			
Poor-grade SAH (WFNS 3–5)	44 (78.6)	15 (68.2)	29 (85.3)	0.184			
Clipping/endovascular‡	20 (35.7)/36 (64.3)	17 (50.0)/17 (50.0)	3 (13.6)/19 (40.9)	**0.009**	0.219	0.050 to 0.956	**0.043**
iHTN duration (days)—mean±SD	11.1 (6.7)	9.1±5.7	13.9±7.2	**0.016**			

Significant p values (<0.05) are written in bold.

* Univariate analysis based on individual tests, as described in the Methods section.

† Multivariate analysis based on logistic regression, with predictor identification via backward selection including only variables contributing significantly to the model in likelihood-ratio testing. Variables were tested for multicollinearity via the assessment of the Variance Inflation Factor with a cut-off of 2.5.

‡ Adjusted ORs for the occlusion modality are in relation to the level clipping, with endovascular treatment being the reference.

CPP, cerebral perfusion pressure; LPR, lactate-to-pyruvate ratio; MAP, mean arterial pressure; PRx, pressure reactivity index; PtiO_2_, brain tissue partial oxygen pressure; Q1, first quartile; Q3, third quartile; SAH, aneurysmal subarachnoid haemorrhage; WFNS, World Federation of Neurological Surgeons grading scale.

**Figure 2:**
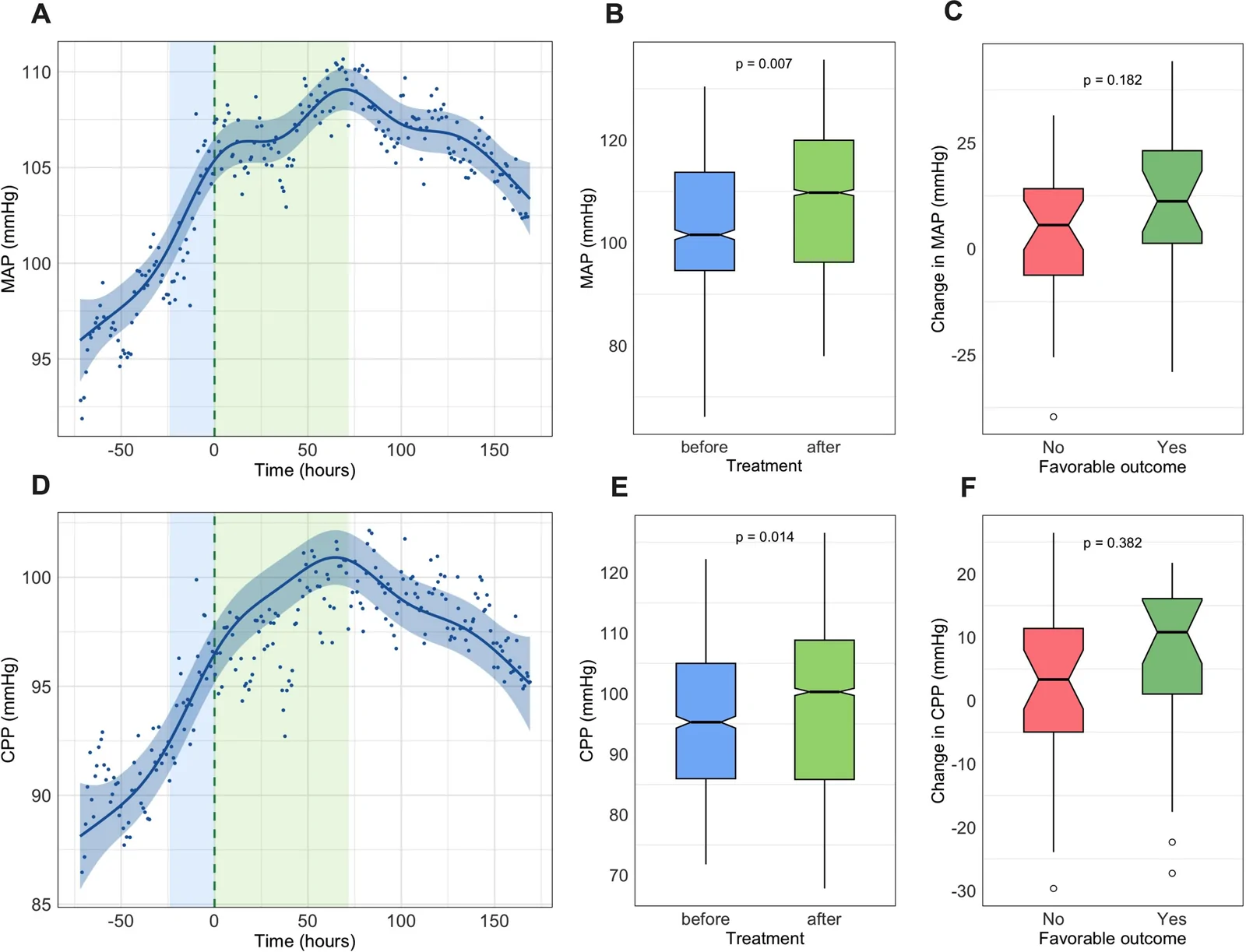
MAP and CPP presented over time around the initiation of DCI treatment and stratified between outcome groups (unfavourable vs favourable outcome). (A and D) Hourly averaged values, over a 10-day time window with an overlayed Generalised Additive Model predicted time courses (blue line) with 95% CIs (darker blue shaded area around the blue line). The green dashed line denotes the beginning of treatment. The blue and green bands represent the 24- and 72-hour time window before and after the beginning of treatment. (B and E) Distributions of averaged pressures depicted as notched box plots, 24 hours before versus 72 hours after treatment initiation. (C and F) Graphical depiction of the magnitude of pressure increase split between dichotomised outcome categories (favourable outcome=mRS 0–3; unfavourable outcome=mRS 4–6). (**A, B, D and E**) Based on data of the patient cohort with DCI and probes optimally located (n=63). (**C and F**) Based on the subset of this cohort for which 1-year outcome was available (n=56). CPP, cerebral perfusion pressure; DCI, delayed cerebral ischaemia; MAP, mean arterial pressure; mRS, modified Rankin Scale.

**Figure 3:**
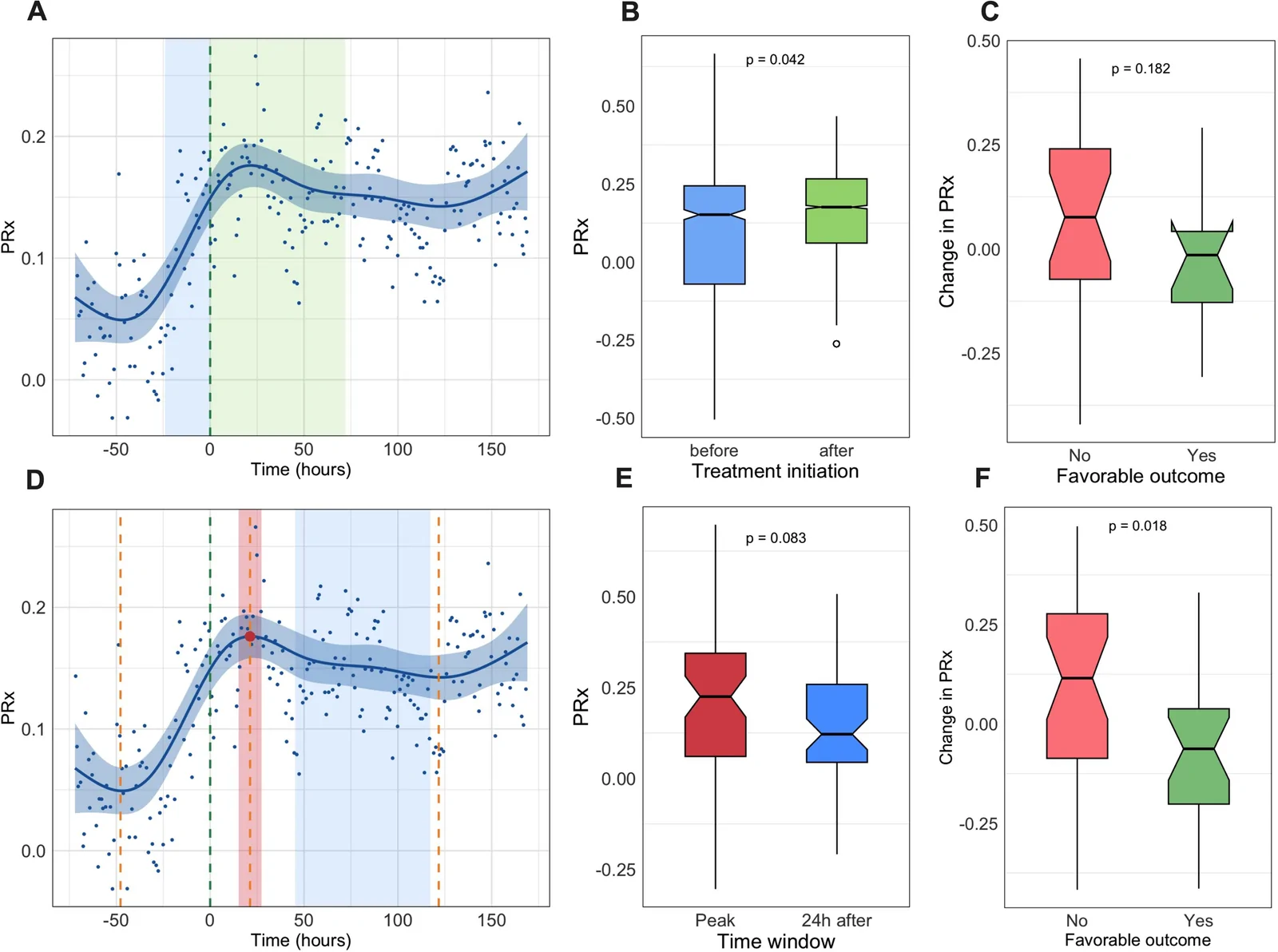
PRx presented over time around the initiation of DCI treatment and stratified between outcome groups (unfavourable vs favourable outcome). (A and D) Hourly averaged PRx, over a 10-day time window with an overlayed Generalised Additive Model predicted time courses (blue line) with 95% CIs (blue shaded area). The green dashed line denotes the beginning of treatment, whereas the yellow dashed lines represent elbows (slope=0). The blue and green bands in (**A**) denote the 24- and 72-hour time window before and after beginning of treatment. The red and blue bands in (**D**) denote a 12-hour segment around the peak and a 72-hour time window starting 24 hours after the peak. Peak average PRx is marked by a red dot. (B and E) Distributions of pooled mean PRx are depicted as notched box plots, 24 hours before versus 72 hours after treatment initiation. (C and F) Graphical depiction of the magnitude of PRx decrease split between dichotomised outcome categories (favourable outcome=mRS 0–3; unfavourable outcome=mRS 4–6). (**A, B, D and E**) Based on data of the patient cohort with DCI and probes optimally located (n=63). (**C and F**) Based on the subset of this cohort for which 1-year outcome was available (n=56). DCI, delayed cerebral ischaemia; mRS, modified Rankin Scale; PRx, pressure reactivity index.

The plotted mean MAP and CPP showed an increase in blood pressure prior to treatment initiation, most likely reflecting permissive hypertension. Temporal trends of MAP and CPP are depicted in figure 2. Once iHTN was initiated, MAP increased by 5.7±15.1 mm Hg and CPP by 10.5±15.6 mm Hg (table 2). The magnitude of MAP and CPP increase did not differ between outcome categories (see figure 2C,F and table 3). Leading up to the DCI event, mean PRx steadily rises, as plotted in figure 3. After the initiation of iHTN, PRx continues to rise and reaches a peak of 0.21±0.24, approximately 25 hours after the beginning of treatment. At this point, PRx stabilises around 0.17±0.16 over a 4-day post-treatment period (see figure 3C,D and table 2). When comparing the difference in PRx (peak vs trough) between outcome groups, the unfavourable outcome group exhibited an increase in PRx of 0.10±0.26, whereas the favourable outcome group demonstrated a decrease of 0.06±0.20 (p=0.018) (see figure 3E).

**Figure 4:**
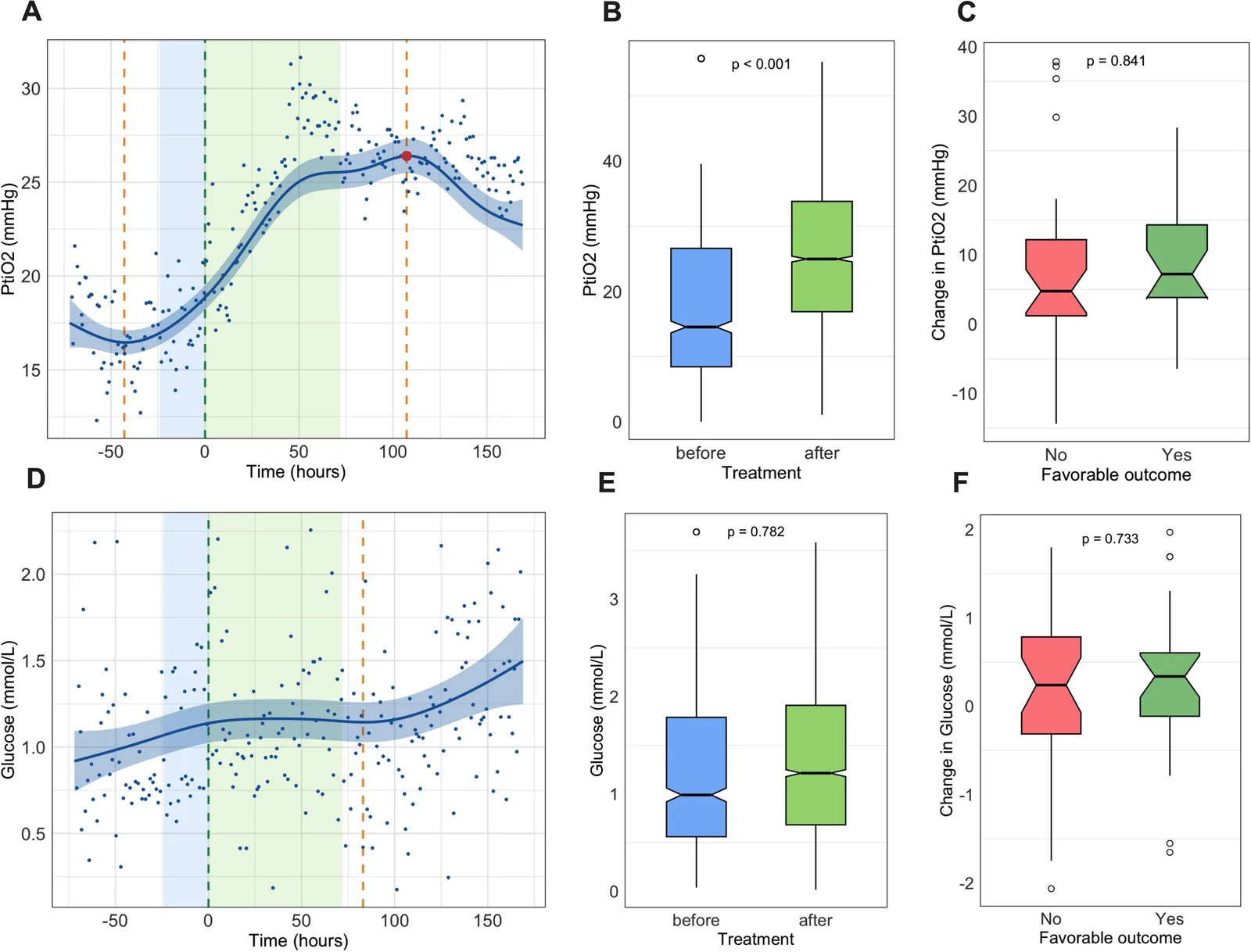
PtiO_2_ (mm Hg) and interstitial glucose concentrations (mmol/L) presented over time around the initiation of DCI treatment and stratified between outcome groups (unfavourable vs favourable outcome). (A and D) Represent hourly median PtiO_2_ and glucose values, over a 10-day time window with an overlayed Generalised Additive Model predicted time courses (blue line) with 95% CIs (blue shaded area). The green dashed line denotes the beginning of treatment, whereas the yellow dashed lines represent elbows (slope=0). The blue and green bands mark a 24- and 72-hour time window before and after the beginning of treatment. (B and E) Distributions of pooled median PtiO_2_ and glucose values are depicted as notched box plots, 24 hours before versus 72 hours after treatment initiation. (C and F) Graphical depiction of the magnitude of PtiO_2_ and glucose increase, split between dichotomised outcome categories (favourable outcome=mRS 0–3; unfavourable outcome=mRS 4–6). (**A, B, D and E**) Based on data of the patient cohort with DCI and probes optimally located (n=63). (**C and F**) Based on the subset of this cohort for which 1-year outcome was available (n=56). DCI, delayed cerebral ischaemia; mRS, modified Rankin Scale; PRx, pressure reactivity index; PtiO_2_, brain tissue partial oxygen pressure.

The time trend of brain tissue oxygen levels is plotted in figure 4A. Median oxygen levels reach a peak around 107 hours after the beginning of treatment. In response to treatment, median PtiO_2_ increased by an average of 6.6±14.3 mm Hg (figure 4B); however, the magnitude of this change did not differ between outcome categories (figure 4C).

Median interstitial glucose levels (mmol/L) are plotted in figure 4C. When comparing baseline with levels 72 hours after treatment, glucose remained stable (figure 4C). A turning point is reached after 83 hours when median glucose levels slowly started rising.

**Figure 5:**
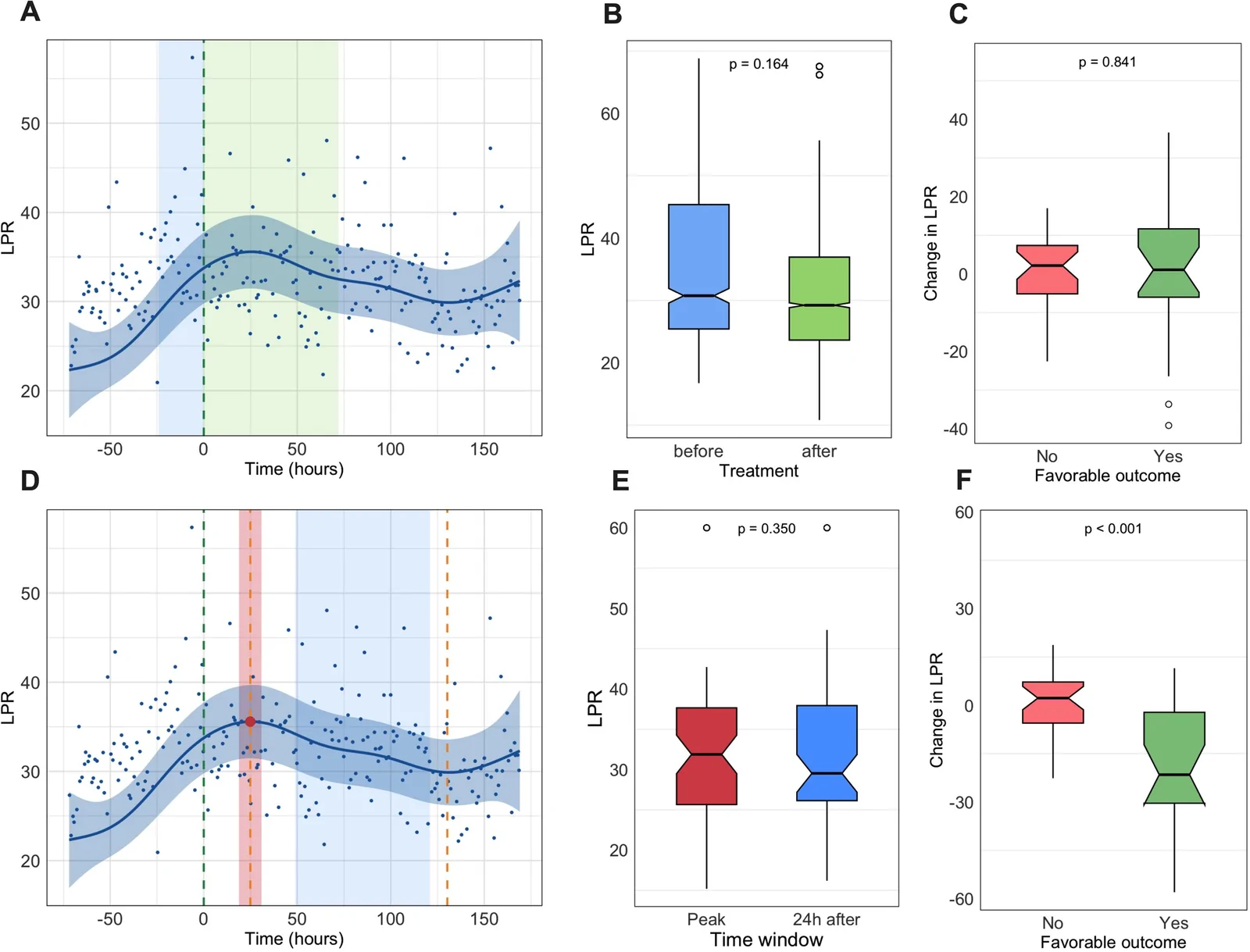
LPR presented over time around the initiation of DCI treatment and stratified between outcome groups (unfavourable vs favourable outcome). (A and D) Represent hourly median values, over a ten-day time window with an overlayed Generalised Additive Model predicted time courses (blue line) with 95% CIs (blue shaded area). The green dashed line denotes the beginning of treatment, whereas the yellow dashed lines represent elbows (slope=0). The blue and green bands in (**A**) denote a 24- and 72-hour time window before and after beginning of treatment. The red and blue bands in (**D**) mark a 12-hour segment around the peak and a 72-hour time window starting 24 hours after the peak. Peak average LPR is marked by a red dot. (B and E) Distributions of pooled median values depicted as notched box plots, 24 hours before versus 72 hours after treatment initiation. (C and F) Graphical depiction of the magnitude of LPR decrease split between dichotomised outcome categories (favourable outcome=mRS 0–3; unfavourable outcome=mRS 4–6). (**A, B, D and E**) Based on data of the patient cohort with DCI and probes optimally located (n=63). (**C and F**) Based on the subset of this cohort for which 1-year outcome was available (n=56). DCI, delayed cerebral ischaemia; LPR, lactate-to-pyruvate ratio; mRS, modified Rankin Scale.

Median LPR over a 10 days time span is plotted in figure 5. Treatment initiation did not induce a significant reduction in LPR when comparing baseline (24 hours) versus 72 hours after treatment (figure 5B). Although median LPR reached a peak after 24 hours, values began to decline thereafter. When comparing peak values with a 72 hours window starting 24 hours after the peak, no significant difference was observed. However, when comparing dichotomised outcome groups, patients developing unfavourable outcomes presented with a mild increase in LPR (1.3±9.1) whereas those rated as favourable outcome demonstrated a decrease (17.6±18.2; p≤0.001). These results are depicted in figure 5F and listed in table 3.

### Treatment effect in multimodal monitoring

A binomial logistic regression model for favourable outcome was constructed, including aneurysm occlusion modality, duration of iHTN and the peak- versus trough-treatment differences in PRx and LPR as predictors. Of the included variables, all but iHTN duration were significantly associated with dichotomised outcome. Removing iHTN duration decreased the AIC from 54.026 to 52.498. Further iterative removal of predictors resulted in an increase in AIC; therefore, the final model retained three predictors. This model demonstrated good explanatory power, with a Nagelkerke R² of 0.33, and the LRT indicated overall model significance (χ²(2)=15.55, p<0.001). After adjusting for aneurysm occlusion modality, reductions in both PRx and LPR following treatment initiation were significantly associated with increased odds of favourable outcome. Specifically, greater reductions in PRx (ie, a more negative difference in PRx) were associated with higher odds of favourable outcome (OR 0.023, 95% CI 0.001 to 0.394, p=0.009). Given that the difference in PRx is a correlation coefficient ranging between −1 and 1, a more interpretable effect is seen per 0.1-unit reduction: each 0.1 decrease in the PRx difference (ie, greater PRx decline) was associated with a 46.5% increase in the odds of a favourable outcome (OR≈1/0.686≈1.46). Similarly, greater reductions in LPR were associated with improved outcomes (OR 0.950, 95% CI 0.904 to 0.998, p=0.042). A 5-unit decrease in the difference in LPR was associated with a 29.3% increase in the odds of a favourable outcome (OR ≈1/0.773≈1.29).

## Discussion

The role of multimodal neuromonitoring beyond the detection of DCI in patients with SAH remains insufficiently explored. In this cohort of severely affected SAH patients, a reduction in PRx as well as LPR in response to DCI treatment was associated with favourable 1-year outcomes. A decrease in PRx reflects an improvement of cerebrovascular reactivity, indicating a better ability of the cerebral vasculature to maintain adequate perfusion pressure. Similarly, a reduction in LPR reflects a shift from anaerobic back to aerobic metabolism, signalling restoration of more normal cellular physiology. While these changes occur in temporal relation to treatment, a direct causal link to specific treatment modalities cannot be established from the current data. Notably, the PRx and LPR responses appeared with some delay (~24 hours), suggesting they may capture more sustained and biologically meaningful improvements than the more immediate but transient increases in PtiO_2_, which reflect oxygen delivery rather than metabolic utilisation. Future studies should examine whether integrating these parameters into treatment algorithms could help guide individualised therapy and improve outcomes after SAH.

This prospective cohort study aimed to identify physiological parameters obtained through MMM that are most strongly associated with successful treatment, that is, resulting in favourable outcome after 1 year. To the best of our knowledge, this aspect has not yet been investigated in the context of iHTN. Its role in monitoring the efficacy of ERT has been explored in subsets of this cohort.^18 19^ In 13 SAH patients undergoing 25 ERT sessions, a significant increase in PtiO_2_ was observed when comparing peak values within a 24-hour post-treatment window to the preceding 24-hour period.^18^ This was accompanied by a statistically significant decrease in LPR, highlighting ERT’s ability to enhance both cerebral blood flow/oxygenation and metabolic function. A similar analysis in 33 SAH patients (also included in this study) across 54 ERT sessions demonstrated an average PtiO_2_ increase of more than 10 mm Hg and an LPR reduction of approximately 15 units.^19^

As a sustained effect on clinical outcomes was expected to require improvements lasting longer than 24 hours, a longer time window of 72 hours after treatment was selected for this study. Multimodal neuromonitoring parameters may respond to treatment in three potential ways. First, a parameter may increase leading up to DCI (eg, LPR) and subsequently decrease following treatment initiation, creating an inverted U-shaped pattern. Second, a parameter may decrease due to DCI development (eg, PtiO_2_) and then increase in response to treatment, resulting in a U-shaped time trend. Third, treatment may have no discernible effect on the temporal profile of a parameter.

In the first two scenarios, pooling of data over a time window of equal length before and after treatment captures changes only if the rate of change differs before and after treatment. Adjusting the time window’s position, shortening or extending it on either side may influence the observed effects. A 24-hour time window before (baseline) and 72 hours after was chosen based on exploratory plotting of local regression lines. We anticipated a steeper increase or decrease over this period, followed by a steadier normalisation after treatment initiation and values stabilising within a normal range for a prolonged duration compared with pretreatment. To evaluate these effects, GAMs were generated up to 1 week after treatment initiation to visualise treatment responses.

### Limitations

This study leverages prospectively collected data with standardised outcome assessments. To enhance data relevance, only patients with neuromonitoring probes placed in or near at-risk brain tissue were included. However, this selective inclusion reduced the sample size, limiting generalisability. Moreover, at the bedside, clinicians are typically unaware of probe positioning relative to hypoperfused areas until imaging is completed, restricting direct clinical applicability.

There are some relevant differences in baseline characteristics between the compared groups. First, the rate of aneurysms treated with surgical clipping was higher in the unfavourable outcome group. Second, patients with a favourable 1-year outcome underwent prolonged iHTN. In this cohort, patients with large intracerebral haematomas or severe brain swelling, indicative of extensive EBI, underwent primary decompressive hemicraniectomy when necessary and were treated with clipping in the same session. Consequently, a selection bias exists toward patients with a lower initial probability of achieving a favourable 1-year outcome. Conversely, selection bias leading to an inverse effect applies to the length of iHTN. Treatment is typically discontinued and spasmolysis withheld if poor clinical outcome is anticipated, leading to shorter durations of treatment. Therefore, any conclusions regarding causal relationships should only be made with the necessary caution.

We acknowledge that the use of iHTN is supported only by observational evidence, and that a randomised trial assessing this treatment yielded negative results.^13^ In addition, ERT has not been assessed in a high-quality randomised controlled trial and is only supported by mostly retrospective cohort studies without a control group. In this study, patients were not stratified according to the type or intensity of endovascular treatment received. Although stratification was attempted, this led to very small subgroups, thereby reducing statistical power. Therefore, in this study, ‘treatment’ should be interpreted as the initiation of therapy according to the algorithm described in the Methods section, and not solely as iHTN.

Pooling data into 24- and 72-hour windows may have diluted pretreatment effects and masked early post-treatment responses, especially in transient interventions like single-session spasmolysis. Additionally, high-frequency physiological data were down-sampled to hourly summaries to align with microdialysis, improving standardisation but reducing real-time interpretability and obscuring short-term fluctuations.

## Conclusion

The clinical utility of multimodal neuromonitoring in aneurysmal subarachnoid haemorrhage extends beyond its role as a diagnostic tool for DCI. In particular, a reduction in PRx or LPR following iHTN was associated with higher odds of favourable 1-year outcome. In contrast, while PtiO_2_ levels demonstrate a clear increase following treatment initiation, the magnitude of this effect is less predictive of treatment success in terms of functional outcome.

## Data Availability

Data are available upon reasonable request.
